# Solar-panel and parasol strategies shape the proteorhodopsin distribution pattern in marine Flavobacteriia

**DOI:** 10.1038/s41396-018-0058-4

**Published:** 2018-02-06

**Authors:** Yohei Kumagai, Susumu Yoshizawa, Yu Nakajima, Mai Watanabe, Tsukasa Fukunaga, Yoshitoshi Ogura, Tetsuya Hayashi, Kenshiro Oshima, Masahira Hattori, Masahiko Ikeuchi, Kazuhiro Kogure, Edward F. DeLong, Wataru Iwasaki

**Affiliations:** 1 Atmosphere and Ocean Research Institute, The University of Tokyo, Chiba, 277-8564 Japan; 2 Department of Natural Environmental Studies, Graduate School of Frontier Sciences, The University of Tokyo, Chiba, 277-8563 Japan; 3 Department of Life Sciences, Graduate School of Arts and Sciences, The University of Tokyo, Tokyo, 153-8902 Japan; 4 Department of Computational Biology and Medical Sciences, Graduate School of Frontier Sciences, The University of Tokyo, Chiba, 277-8561 Japan; 5 Department of Bacteriology, Faculty of Medical Sciences, Kyushu University, Fukuoka, 812-8582 Japan; 6 Center for Omics and Bioinformatics, Graduate School of Frontier Sciences, The University of Tokyo, Chiba, 277-8561 Japan; 7 Graduate School of Advanced Science and Engineering, Waseda University, Tokyo, 169-8555 Japan; 8 Center for Microbial Oceanography: Research and Education, University of Hawaii, Honolulu, HI 96822 USA; 9 Department of Biological Sciences, Graduate School of Science, The University of Tokyo, Tokyo, 113-0032 Japan

## Abstract

Proteorhodopsin (PR) is a light-driven proton pump that is found in diverse bacteria and archaea species, and is widespread in marine microbial ecosystems. To date, many studies have suggested the advantage of PR for microorganisms in sunlit environments. The ecophysiological significance of PR is still not fully understood however, including the drivers of PR gene gain, retention, and loss in different marine microbial species. To explore this question we sequenced 21 marine Flavobacteriia genomes of polyphyletic origin, which encompassed both PR-possessing as well as PR-lacking strains. Here, we show that the possession or alternatively the lack of PR genes reflects one of two fundamental adaptive strategies in marine bacteria. Specifically, while PR-possessing bacteria utilize light energy (“solar-panel strategy”), PR-lacking bacteria exclusively possess UV-screening pigment synthesis genes to avoid UV damage and would adapt to microaerobic environment (“parasol strategy”), which also helps explain why PR-possessing bacteria have smaller genomes than those of PR-lacking bacteria. Collectively, our results highlight the different strategies of dealing with light, DNA repair, and oxygen availability that relate to the presence or absence of PR phototrophy.

## Introduction

Proteorhodopsin (PR) plays a fundamental role in marine ecosystems as a light-driven proton pump protein that converts light energy to proton motive force [1]. While this proton motive force is sufficient to generate ATP [2, 3], PR-possessing (PR+) prokaryotes may also utilize light energy to uptake organic compounds [4]. Pioneering studies on PR+ marine Flavobacteriia showed that light fosters bacterial growth [5] and that their PR expression is likely coupled with carbon assimilation through anaplerotic inorganic carbon fixation [6, 7]. PR-mediated photoheterotrophy is broadly distributed among various groups of marine prokaryotes in which Alphaproteobacteria, Gammaproteobacteria, and Flavobacteriia are the major groups, being consistent with the estimated physiological benefits of having PR. Recent culture-independent surveys showed that PR genes can occur in up to 80% of prokaryotes in the marine photic zone [8], and their RNA and protein expression levels are high [4, 9]. Overall, accumulating evidence suggests that possessing PR is generally advantageous to an organism in sunlit marine microbial ecosystems.

On the other hand, the growing understanding of PR function provokes another fundamental question—if the possession of PR is so advantageous acting as bonus “solar panels” for microbes, why are there so many PR-lacking (PR−) prokaryotes in the marine photic zone [3]. Comparative genomics is a potentially useful approach for answering such questions because genomes fundamentally reflect microbial ecophysiology [10–13]. That is, systematic differences between PR− and PR+ prokaryote genomes might provide clues about differences in the lifestyles of these microbes. Genomic differences revealed in a previous study showed that PR− Flavobacteriia have significantly larger genomes than PR+ Flavobacteriia, although the ecophysiological reasons for this phenomenon remains enigmatic [11].

In this study, we sequenced 21 marine Flavobacteriia genomes and conducted comparative genomic analysis of 41 PR− and 35 PR+ marine Flavobacteriia. From a methodological perspective, our analysis was performed to fulfil two prerequisite conditions for successfully discovering systematic differences between different types of genomes [14, 15]. First, to attenuate strain-specific signals and achieve sufficient statistical power, a sufficiently large number of genomes that were not strongly biased within a single type are required. Second, genomes that belong to each different type need to be phylogenetically dispersed. Otherwise, genomic differences due to phylogenetic constraints (i.e., an effect that phylogenetically closely related species tend to have similar genomes just because they share a common ancestor) as opposed to ecophysiological adaptations, will bias the analysis.

## Materials and methods

### Sample preparation and genome sequencing

Supplementary Table S1 shows the summary of 21 marine Flavobacteriia strains whose genomes were sequenced in this study. Seven *Polaribacter* (*P. butkevichii* KCTC 12100^T^, *P. gangjinensis* KCTC 22729^T^, *P. glomeratus* ATCC 43844^T^, *P. sejongensis* KCTC 23670^T^, *P. reichenbachii* KCTC 23969^T^, *P. porphyrae* NBRC 108759^T^, and *P. filamentus* ATCC 700397^T^) and six *Nonlabens* (*N. agnitus* JCM 17109^T^, *N. arenilitoris* KCTC 32109^T^, *N. sediminis* NBRC 100970^T^, *N. spongiae* JCM 13191^T^, *N. tegetincola* JCM 12886^T^, and *N. xylanidelens* DSM 16809^T^) type strains were provided by the NITE Biological Resource Center (NBRC), Japan Collection of Microorganisms (JCM), American Type Culture Collection (ATCC), Korean Collection for Type Cultures (KCTC), and Deutsche Sammlung von Mikroorganismen und Zellkulturen GmbH (DSMZ). The other eight strains were isolated from environmental samples in 2009 [3]: four strains from surface seawater at Western North Pacific Station S (30°40′N, 138°00′E) during cruise KT-09-11 of the R/V ‘Tansei Maru’ (Atmosphere and Ocean Research Institute, The University of Tokyo and Japan Agency for Marine-Earth Science and Technology (JAMSTEC)) (*Aureicoccus marinus* SG-18^T^, *Tenacibaculum* sp. SG-28, *Tenacibaculum* sp. SZ-18, and *Gilvibacter* sp. SZ-19), one strain from surface seawater at Western North Pacific Station S1 (30°11′N, 145°05′E) during cruise MR10-01 of the R/V ‘Mirai’ (JAMSTEC) (*Aureitalea marina* NBRC 107741^T^), two strains from sea ice in Saroma-ko Lagoon (44°07′N, 143°58′E) (*Polaribacter* spp. SA4-10 and SA4-12), and one strain from surface seawater at Sagami Bay Station P (35°00′N, 139°20′E) during cruise KT-09-11 (*Winogradskyella* sp. PC-19). All strains were cultivated using half strength ZoBell’s 2216E medium.

Genomic DNA samples were extracted by the standard phenol–chloroform method [16]. Genomes of two strains were sequenced using a 454 FLX+ System (Roche) and an Ion PGM System (Thermo Fisher Scientific) and assembled using the Newbler assembler v2.7 software (Roche). Genomes of 11 strains were sequenced using a 454 FLX+ System and a MiSeq (Illumina) platform and assembled using the Newbler assembler v2.7 software. Genomes of the other eight strains were sequenced using a PacBio RS II (Pacific Biosciences) instrument and assembled using Sprai v0.9.5.1.3 (http://zombie.cb.k.u-tokyo.ac.jp/sprai/) and subsequent manual curation. All sequencing was performed by following manufacturers' protocols, and all assembling steps were performed using default parameters.

### Data set preparation and assessment of genome completeness

We downloaded 55 genomes of marine Flavobacteriia from the NCBI RefSeq database [17] (Supplementary Table S2). During the quality check of the sequenced genomes, we found that several scaffolds of *P. sejongensis* KCTC 23670^T^ and *P. reichenbachii* KCTC 23969^T^ genomes were likely to be contaminated. We randomly selected 10 protein-coding sequences (CDSs) from the six scaffolds that coded CDSs and identified their origins by sequence similarity searches against the UniProt database [18] (downloaded in April 2017, results in Supplementary Table S3). The origin of each scaffold was consistently estimated at the genus level, and only the largest scaffold from each genome was concluded to be from the *Polaribacter* strains. We assessed the completeness of these two genomes after removing the other scaffolds using Benchmarking Universal Single-Copy Orthologs (BUSCO) version 3.0.0 [19] and a 443 orthologue data set that is conserved in the class Bacteroidetes, and obtained high scores (97.7% for *P. sejongensis* KCTC 23670^T^ and 98.4% for *P. reichenbachii* KCTC 23969^T^).

The completeness of all 76 genomes was also assessed using BUSCO on the Bacteroidetes orthologue data set. The scores averaged 98.0%, and the completeness of five genomes was <95.0%. The lowest BUSCO score was that of *Salinibacter ruber* DSM 13855 ^T^, which had acquired many genes from hyperhalophilic archaea [20]. Excluding the five genomes with <95.0% completion did not affect the conclusions of this study.

### Functional annotation of genes

All 76 genomes were annotated through the following procedure. Ribosomal and transfer RNA genes were annotated using RNAmmer v1.2 [21] and tRNAscan-SE v1.3.1 [22], respectively, with their default settings. Subsequently, we masked the rRNA and tRNA gene sequences with “N” and predicted CDSs using Prodigal v2.50 [23] at default settings.

The functional annotation of the 258,135 CDSs was performed by eggNOG-mapper [24] and the bactNOG data set in the eggNOG database version 4.5 [25], by adopting the DIAMOND option for mapping and by setting the taxonomic scope to Bacteroidetes. This approach resulted in functional annotation of 184,623 (71.5%) of the CDSs to 14,361 eggNOG orthologue groups, excluding function-unknown orthologue groups (i.e., groups whose annotations contained any of the terms “NA”, “unknown”, or “DUF”).

Amino-acid sequences of CDSs that were annotated to the rhodopsin orthologue group (ENOG05CSB) were aligned using MAFFT version 7.212 [26] with the linsi algorithm and default parameters. The alignments were curated using trimAl version 1.2 [27] with the option “–gt 1”. The best substitution model of each alignment was selected using prottest3 [28]. The maximum-likelihood method was performed using RAxML version 7.2.8 [29] and 1000 bootstrap replicates. The other settings were set to their default values. Phylogenetic classification of rhodopsins as PR, Na^+^-pumping rhodopsin (NaR), and Cl^−^-pumping rhodopsin (ClR) genes was conducted as described in our previous study [30].

### Reconstruction of the genomic phylogenetic tree

As outgroups, genomes of two strains of the class Bacteroidetes (*Cytophaga hutchinsonii* ATCC 33406^T^ and *Salinibacter ruber* DSM 13855^T^) were additionally downloaded from the NCBI RefSeq database. The prediction and annotation of their CDSs were conducted in the same manner as the other genomes. We selected 155 ENOG orthologue groups so that each genome contained exactly one CDS that was annotated to each of those orthologue groups. Their amino-acid sequences were aligned using MAFFT and curated using trimAl as described above. The best substitution model of each alignment was selected by using prottest3. The alignments of 155 eggNOG orthologue groups were concatenated and subjected to phylogenetic tree reconstruction using RAxML with the best substitution model for each protein column and 1000 bootstrap replicates. The other settings were set to their default values.

### Genome size and gene content analysis

The difference in the sizes of the PR− and PR+ genomes was statistically evaluated by applying Student’s *t*-test to the total scaffold sizes of the two groups. Orthologue group distributions that were biased in PR− or PR+ genomes were identified by applying the Brunner–Munzel test [31] to the numbers of CDSs in each of the 14,361 eggNOG orthologue groups. To correct for multiple testing, Storey’s approach [32] was used with a cut-off false discovery rate of 0.05.

For the orthologue group distribution analyses across different phyla, the eggNOG 4.5 database [25] and Microbial Genome Database for Comparative Analysis (MBGD) database, updated on 2015 April, was used.

### Experimental analysis of DUF2237

The DUF2237 gene of *Synechocystis* sp. PCC 6803-P (i.e., *slr*1628) [33] was inactivated by replacing it with a chloramphenicol resistance cassette. A DNA sequence that contained the region that is 500-bp upstream of the DUF2237 gene, a chloramphenicol resistance cassette, and 500-bp downstream of DUF2237 was artificially synthesized and inserted into a pEX-A2 vector (Eurofins Genomics). Knockout strains were generated by transforming this plasmid into PCC 6803-P cells, growing these cells at 30 °C under continuous white light with an intensity of 50 μmol m^−2^ s^−1^, and selecting colonies on plates with BG-11 medium [34] that contains 20 μg m1^−1^ chloramphenicol. Because PCC 6803-P cells contain multiple genomes in each cell, the segregation between the wild-type and DUF2237-knockout genomes was examined by PCR with DUF2237-upstream (5’-AATCTCTGCTAGGTTTGG-3’) and DUF2237-downstream (5’-AACTCTGGTAGCTGTTCC-3’) primers after 3 days of growth on the BG-11 plates.

For the phototaxis assay, wild-type and DUF2237-knockout cells were collected in the exponential phase, suspended in BG-11 liquid medium at an optical density of 0.1, and spotted onto 1.5% agarose BG-11 plates four times per strain. The spotted plates were incubated under unidirectional white light with an intensity of 22 μmol m^−2^ s^−1^ at 30 °C for 7 days, and the distances of colony movements were measured.

### Analysis of RNR gene classes

To identify the classes of RNR genes, all CDSs that were annotated with the ENOG05BZH were fed into domain-level annotation using the NCBI conserved domain search [35]. For phylogenetic analysis of RNR genes, RNR genes of *Lactobacillus leichmannii* (GenBank: AAA03078) and *Escherichia coli* H736 (GenBank: EGI11882) were downloaded from GenBank to serve as representatives of class II and class I genes, respectively. CDSs were aligned, and the alignments were curated by the same methods described above. The best-fit substitution model was selected using prottest3 at its default settings. The maximum-likelihood method was performed using RAxML and 1000 bootstrap replicates. The other settings were set to their default values.

### Analysis of Tara Oceans data set

The Tara Oceans data set, containing gene abundance FPKM value, oxygen concentration, and sampling depth data, was downloaded from http://ocean-microbiome.embl.de/companion.html [36]. Correlation analysis was conducted using the “psych” package in R (https://pbil.univ-lyon1.fr/CRAN/web/packages/psych/). Curve fitting was done using locally weighted scatterplot smoothing with its default options.

## Results

### Marine Flavobacteriia genome sequencing and data set preparation

To obtain a large, unbiased, and polyphyletic (phylogenetically dispersed) genomic data set, genomes of 21 marine Flavobacteriia strains were sequenced. These strains contained seven *Polaribacter* type strains, six *Nonlabens* type strains, and eight strains that were isolated from Saroma-ko Lagoon (Hokkaido, Japan), Sagami Bay (Kanagawa, Japan), and the western North Pacific Ocean (Supplementary Table S1). We subsequently downloaded 55 genomes of marine Flavobacteriia from the NCBI RefSeq database [17] and constructed a genomic data set of 76 marine Flavobacteriia strains, 41 and 35 of which were PR− and PR+ strains, respectively (Supplementary Table S2; their sampling sites are shown in Supplementary Fig. S1). All genomes were subjected to in-house annotation of their ribosomal RNAs, transfer RNAs, and CDSs. To evaluate the quality of the 76 genomes, their completeness was estimated using BUSCO (version 3.0.0) software [19]. All but five genomes were >95% complete by this metric. Excluding those five genomes from the analyses did not affect the conclusions of this study.

### Functional annotation and confirmation of polyphyletic PR distribution

The CDSs were functionally annotated using eggNOG-mapper [24] and the bactNOG data set in the eggNOG database [25]. Among the 258,135 CDSs in total, 71.5% were assigned to any eggNOG orthologue group by ignoring function-unknown groups. We further classified the CDSs that were assigned to the rhodopsin orthologue group (ENOG05CSB) as PR, NaR, and ClR genes by phylogenetic analysis (Supplementary Fig. S2). Whereas all NaR-possessing strains had additional PR genes, two ClR-possessing strains (*Nonlabens spongiae* JCM 13191^T^ and *Psychroserpens* sp. Hel_I_66) were revealed to lack PR genes. We treated these two ClR-possessing strains as PR+ strains in the following analyses because the inward Cl^−^-pumping activity of ClR also generates membrane potential; however, the conclusions were not affected even if they were treated as PR− strains.

**Fig. 1:**
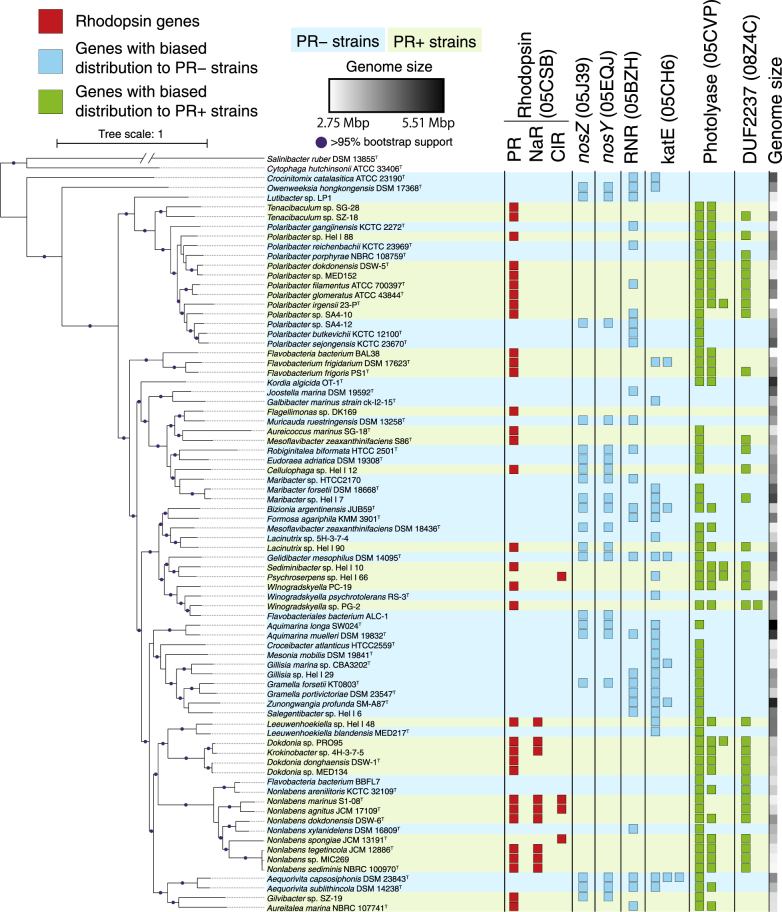
Phylogenetic tree and distributions of genes biased in PR− or PR+ genomes. (Left) A maximum-likelihood genomic tree based on 155 CDSs that were conserved across the 76 marine Flavobacteriia genomes. The closed circles indicate branches with 95% bootstrapping support. Yellow and purple background colours indicate PR− and PR+ strains, respectively. The tree was visualized using iTol v 3.3.2 [71]. (Right) The number of genes coded by each genome is represented by the numbers of closed squares. Red: Rhodopsin genes. Light blue: eggNOG orthologue groups that showed distributions that were particularly biased in PR− genomes. Light green: those particularly biased in PR+ genomes. For RNR (05BZH) and photolyase (05CVP) orthologs, one gene in each strain is not shown because all strains except for *Lutibacter* sp. LP1 possess at least one copy of those orthologs. Note that distributions of genes for the synthesis and transport of APEs are shown in Fig. 5. The genome size of each strain was indicated in grey scale

We then reconstructed a genomic phylogenetic tree of the 76 marine Flavobacteriia strains by applying the maximum-likelihood method to the concatenated protein sequence data set of 155 conserved CDSs that were present in each strain in exactly one copy. To root the tree, genomes of two strains of the phylum Bacteroidetes were added to the data set as outgroups. We confirmed that the PR− and PR+ strains had polyphyletic distributions on the reconstructed phylogenetic tree, fulfilling the second condition for a comparative genomic study (Fig. 1).

### Detection of genes significantly biased in either PR− or PR+ genomes

**Fig. 2:**
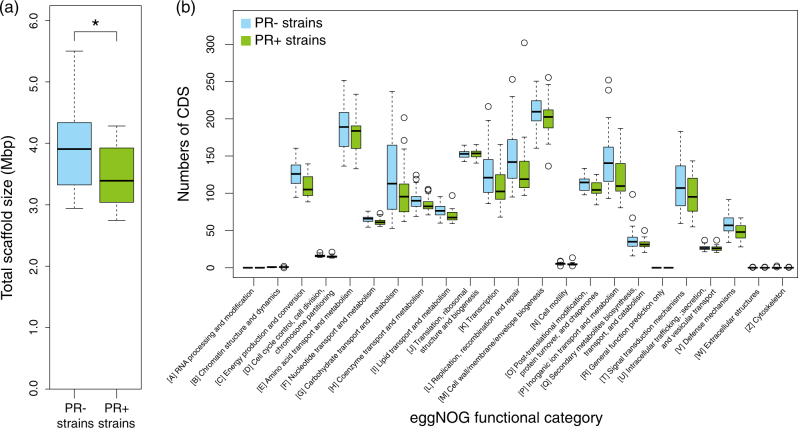
Genome sizes and quantities of CDSs in PR− and PR+ marine Flavobacteriia. **a** Total scaffold sizes of PR− (blue) and PR+ (green) genomes. The bottom, central line, and top of the box plots represent the first, second, and third interquartile ranges (IQR), respectively. Whiskers represent the lowest and highest values within 1.5 × IQR from the first and third quartiles, respectively. **b** Quantities of CDSs in each eggNOG functional category in the box plot drawn in the same manner. Circles represent outliers beyond the whiskers

We first compared the genome sizes of PR− and PR+ marine Flavobacteriia strains. As consistent with previous findings [11], the PR− genomes were significantly larger than the PR+ genomes (*p*-value = 4.7E-3, Figs. 1, 2a). To further investigate the ecophysiological background that caused this difference in genome size, we compared their CDS numbers in each eggNOG functional category [25] (Fig. 2b). We discovered that except for several categories that are generally rare in bacteria, the numbers of CDSs were consistently larger in the PR− genomes than in the PR+ genomes, regardless of their functional categories. This result suggests that the observed genome size difference is not due to acquisitions (in the PR− strains) or losses (in the PR+ strains) of gene sets involved in specific metabolic and/or cellular systems but rather due to net acceleration of genome size expansion (in the PR− strains) or reduction (in the PR+ strains).

Next, we investigated if there are specific eggNOG orthologue groups that had particularly biased distributions. A statistical test detected 86 and 43 (129 in total) orthologue groups whose distributions were significantly biased in the PR− and PR+ genomes, respectively (q-value < 0.05, Supplementary Tables S4 and S5). Except for the trivial case of the PR gene itself, the most significant case was the enrichment of the beta-carotene dioxygenase (*blh*) gene (ENOG05FTR) in the PR+ genomes. This result is quite reasonable because the *blh* gene is involved in the synthesis of retinal, the chromophore of PR.

One unexpected finding was that most of the genes involved in anaplerotic inorganic carbon fixation were not included in the orthologue groups that showed biased distributions in the PR+ genomes (except for the *sbtA* gene (ENOG05EGC), Supplementary Fig. S3). In a previous study, PR+ Flavobacteriia were argued to have significantly more genes involved in anaplerotic inorganic carbon fixation [11] for PR-coupled carbon fixation and light-promoted growth [6, 7]. We assume that the previously observed larger proportion of those genes in PR+ genomes might be due to insufficient data size analyzed. Instead, based on the universal occurrence pattern of those genes, we assume that the fixation of inorganic carbonic acid by anaplerotic carbon fixation would be a common feature among marine Flavobacteriia.

### Experimental analysis of a function-unknown gene strongly biased in PR+ genomes

**Fig. 3:**
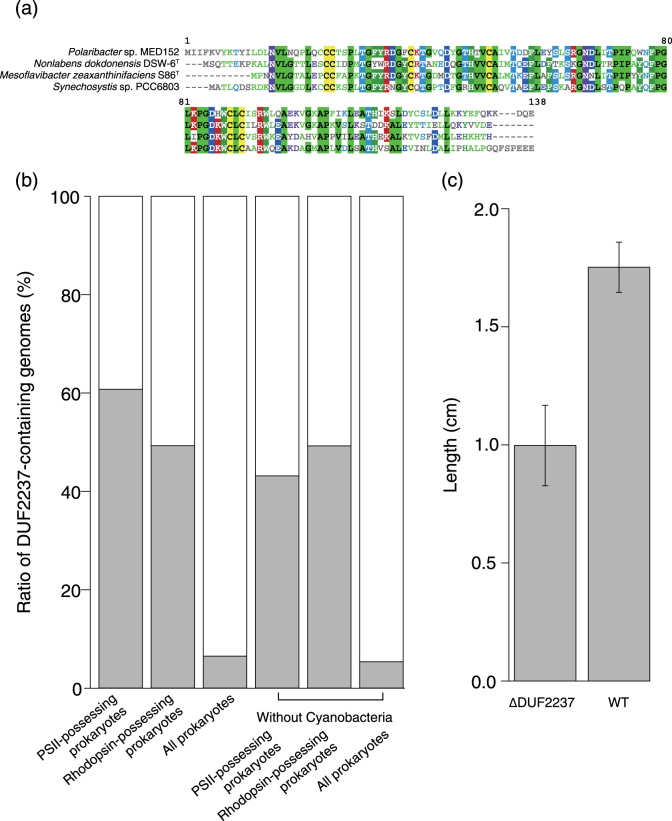
Analysis of the DUF2237 gene. **a** Multiple alignment of DUF2237 amino-acid sequences of Flavobacteriia strains and *Synechocystis* sp. PCC6803. Amino acids with background colours indicate residues with >50% consensus. The multiple alignment was conducted by using MAFFT with the linsi algorithm and its default options and was visualized by using MView [72]. **b** Biased distribution of DUF2237 genes to phototrophs. The bar chart represents the ratios of DUF2237-possessing strains in the PSII-possessing prokaryotes (*n* = 51), rhodopsin-possessing prokaryotes (*n* = 52), all prokaryotes (*n* = 547), PSII-possessing non-cyanobacterial prokaryotes (*n* = 20), rhodopsin-possessing non-cyanobacterial prokaryotes (*n* = 44), and all non-cyanobacterial prokaryotes (*n* = 515). The data were obtained from the MBGD database. **c** Phototaxis assay of the DUF2237 gene knockout strain of *Synechocystis* sp. PCC6803-P. The bar graph shows the distances of colony movements from the spotted points under unidirectional light. Four replicated experiments were conducted for each strain. Statistical significance was examined by Student’s *t*-test (*p*-value = 2.9E-4)

The orthologue group that showed the second most biased distribution contained the DUF2237 genes (q-value = 3.9E-10), which were function-unknown genes that were enriched in the PR+ genomes (Fig. 1 and Supplementary Table S5). Using the MBGD [37], we found that DUF2237 genes (MBGD ID 4444) are broadly distributed across 11 phyla, and many Cyanobacteria, phototrophic bacteria, and rhodopsin-containing Euryarchaeota have this gene. The sequence of the DUF2237 gene is highly conserved across different phyla (Fig. 3a). MBGD analysis showed that DUF2237 is possessed by 72% and 66% of prokaryotes that have photosystem II (*pufM*/*psbA*/*pufL*, MBGD ID 2841) and rhodopsin genes (MBGD ID 22185 and 4672), respectively, whereas only 17% of all prokaryotes have DUF2237 (Fig. 3b). This bias was not just because Cyanobacteria tend to have DUF2237 (i.e., phylogenetic constraint); we confirmed that excluding Cyanobacteria did not diminish the observed bias (Fig. 3b). These observations strongly suggested that DUF2237 has a widely conserved function that is related to general phototrophy.

To experimentally confirm the functional importance of DUF2237, we knocked its gene out of *Synechocystis* sp. PCC6803-P [38]. We selected this cyanobacterial strain because it has a DUF2237 gene and methods to manipulate its genome are well established. The DUF2237-knockout strain did not show any apparent difference in proliferation speed and other phenotypes under standard laboratory culture conditions; however, in phototaxis assays, the DUF2237-knockout strain showed significantly less movement than the wild-type strain, which exhibits positive phototaxis under unidirectional white light (*p*-value = 2.9E-4, Fig. 3c). This result is consistent with the strong correlation between the presence of DUF2237 and phototrophy because phototaxis should be beneficial to organisms that utilize light. Although cyanobacterial phototaxis is a phenotype in which many proteins are involved (e.g., light sensing, signal transduction, transcriptional regulation, and pilus formation proteins) [38–40] and further analyses are required to clarify the molecular basis of the DUF2237 function, this result proves that our comparative genomics approach is powerful enough to find genes that reflect microbial ecophysiology.

### Proximity analysis of genes biased in PR**−** or PR+ genomes

**Fig. 4:**
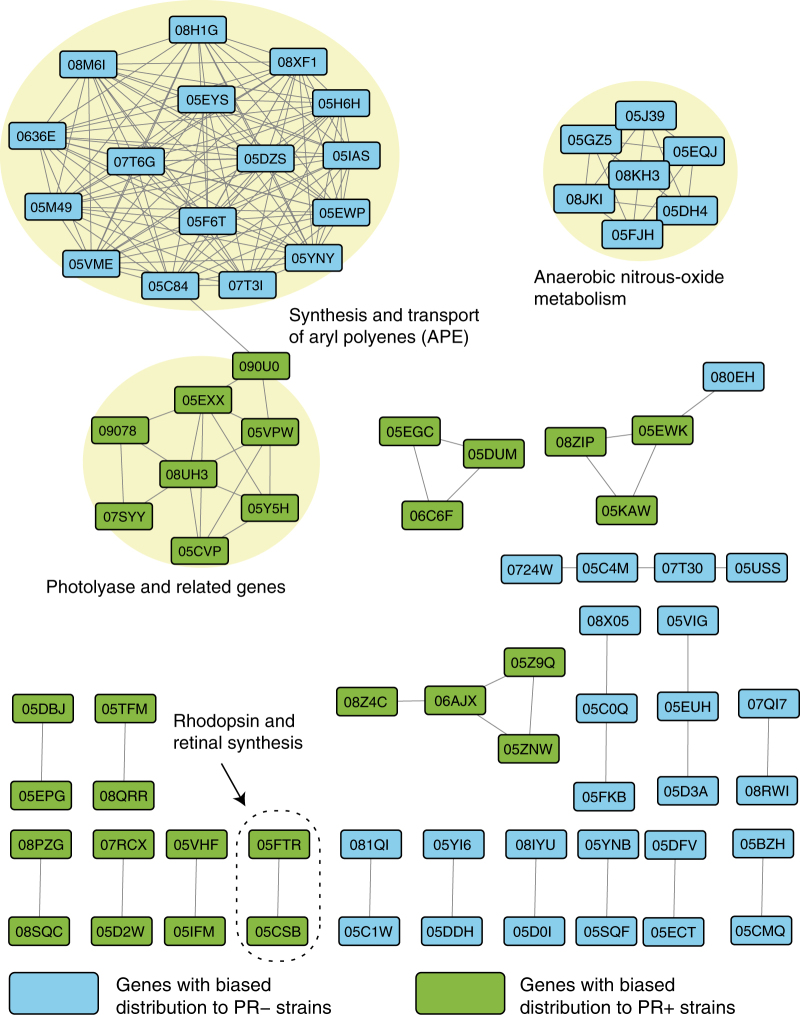
Gene proximity network of orthologue groups that showed biased distributions. Boxes in light blue and light green represent eggNOG orthologue groups that are biased in the PR− and PR+ genomes, respectively. Note that 49 of the 129 orthologue groups showed no proximal relations and are absent from this figure. Three large clusters are indicated by the light-yellow ellipses. The PR and *blh* genes are indicated by the ellipse with a dashed border. Gene annotations are available in Supplementary Tables S4 and S5

We conducted gene proximity analysis of the 129 orthologue groups that showed biased distributions in PR– or PR+ strains because genes that are near each other in genomes likely have related functions [41]. A gene proximity network was constructed by connecting any orthologue group pair that are located within 20 kb of each other in at least 10 genomes in our data set (Fig. 4). A typical example of such proximal relation was seen between the rhodopsin and *blh* genes, which are often adjacently coded for concerted expression [42]. We note that the two ClR-possessing PR– strains code the *blh* genes next to their ClR genes.

Three large clusters were formed in the gene proximity network. Among them, two clusters were composed of genes that were enriched in the PR− genomes: the first was composed of genes for anaerobic nitrous oxide metabolism, and the second was composed of genes for synthesis and transport of aryl polyenes (APEs). The third cluster was composed of photolyase and photolyase-related genes, which were enriched in the PR+ genomes. These three large clusters were assumed to especially reflect lifestyles to which PR− and PR+ Flavobacteriia species have adapted.

### Signs of adaptation of PR− Flavobacteriia to anaerobic conditions

Despite a predominance of function-unknown genes in the 129 orthologue groups that showed biased distributions, we discovered one interesting trend therein: the genes enriched in the PR− genomes showed several signs of adaptation to microaerobic or anaerobic conditions although Flavobacteriia species are usually considered to be (strictly) aerobic [43].

The PR− genomes coded significantly more nitrous oxide reductase (*nosZ*, ENOG05EQJ) and nitrous oxide metabolism (*nosY*, ENOG05J39) genes than the PR+ genomes (Fig. 1, q-value = 3.7E-2 and 3.7E-2, respectively). These genes, which were members of the first cluster that was formed in the gene proximity network (Fig. 4), function in bacterial anaerobic N_2_O respiration [44, 45], which uses nitrous oxide as a terminal electron acceptor at reduced oxygen concentrations [46]. Second, the PR− genomes had more class II ribonucleotide reductase (RNR) genes (ENOG05BZH) (Fig. 1, q-value = 9.9E-5). RNR proteins catalyze the synthesis of deoxyribonucleotides from ribonucleotides and are grouped into three classes according to their subunit types [47]. NCBI conserved domain searches [35] and a phylogenetic analysis (Supplementary Fig. S4) showed significant enrichment of the class II RNR genes in the PR− genomes (PR−: 23/41, PR+: 2/35) occurred. Class II RNRs do not depend on oxygen for their catalytic function, whereas class I RNRs function under aerobic conditions [47]. A catalase gene, *katE* (ENOG05CH6), was also enriched in the PR− genomes (Fig. 1, q-value = 4.9E-3). This gene was reported to modulate reactive oxygen stress when cells that usually live in anaerobic conditions are exposed to oxygen. Expression of *katE* increases under anaerobic conditions in *E. coli* [48], and the *katE* protein is the only H_2_O_2_-removing enzyme that is present in an obligate anaerobic Bacteroidetes, *Bacteroides thetaiotaomicron* [49, 50]. In addition, the PR− genomes almost always had *cbb*_3_-type cytochrome oxidase genes (ENOG05EUH), whereas the PR+ genomes did not (Supplementary Fig. S5, q-value = 1.6E-2). The *cbb*_3_-type cytochrome oxidases have a very high affinity for O_2_ so that their organisms can respire under microaerobic conditions [51], and they should enable Flavobacteriia to survive in transiently low-O_2_ microniches [52].

### Enrichment of ultraviolet (UV)-screening pigment synthesis genes in PR− genomes

**Fig. 5:**
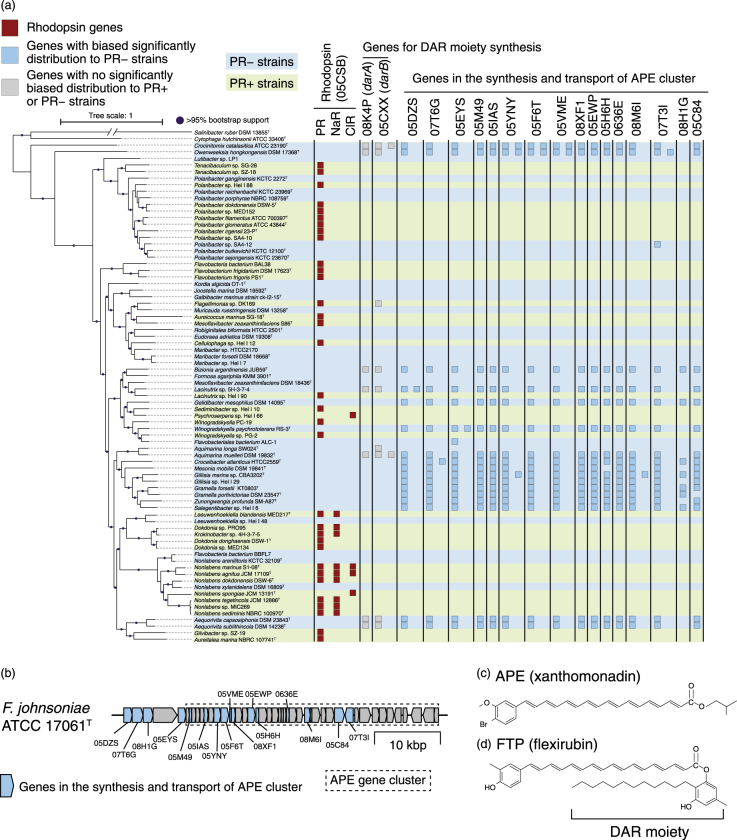
Analysis of genes involved in the synthesis and transport of APEs. **a** Distributions of genes involved in synthesis and transport of APEs and DAR. The genomic phylogenetic tree is from Fig. 1. The closed circles indicate branches with 95% bootstrapping support. Yellow and purple background colours indicate PR− and PR+ strains, respectively. Number of genes coded by each genome is illustrated by the number of closed squares. Red: rhodopsin genes. Grey: eggNOG orthologue groups involved in the synthesis of the DAR moiety (05CXX: *darB* and 08K4P: *darA*) and did not show biased distributions. Light blue: eggNOG orthologue groups in the cluster related to the synthesis and transport of APEs (05DZS: phenylacetate-CoA ligase, 07T6G: acyl-coenzyme A 6-aminopenicillanic acid acyltransferase, 05EYS: glycosyl transferase, family 2, 05M49: dehydratase, 05IAS: outer membrane lipoprotein carrier protein LolA, 05YNY: synthase, 05F6T: synthase, 05VME, acyl carrier protein, 08XF1: NA, 05EWP: synthase, 05H6H: thioesterase, 0636E: flexirubin-type pigment biosynthesis acyl carrier protein, 08M6I: lipid A biosynthesis acyltransferase, 07T31: 5’-nucleotidase, 08H1G: phospholipid glycerol acyltransferase, and 05C84: histidine ammonia-lyase) that showed distributions that were significantly biased in the PR– genomes. **b** Syntenic map of the APE gene cluster of *F. johnsoniae* ATCC 17061^T^. Pentagons represent genes, and pentagon lengths are proportional to the gene lengths. Genes in the cluster related to the synthesis and transport of APEs in Fig. 3 are shown in light blue. The previously reported APE gene cluster of *F. johnsoniae* ATCC 17061^T^ (between *Fjoh_1080* and *Fjoh_1115* genes) is represented by a rectangle with a dashed border. **c** Structure of a representative APE molecule (xanthomonadin). **d** Structure of a representative FTP molecule (flexirubin)

The second cluster in the gene proximity network contained 16 genes for the synthesis and transport of APEs and was enriched in the PR− genomes (Fig. 4). Most notably, almost all genes in this cluster were not only significantly but also exclusively found in the PR− genomes (Fig. 5a). The genes in this cluster corresponded well to those previously reported in an APE-producing gene cluster in the *Flavobacterium johnsoniae* ATCC 17061^T^ genome [53] (Fig. 5b). These data strongly suggested that production of APEs is a unique feature of PR− marine Flavobacteriia.

APEs (Fig. 5c) protect bacterial cells from UV and visible light by localizing to outer membranes [54, 55]. This localization to outer membranes contrasts with the localization of carotenoids to inner membranes [56] but resembles that of scytonemin, a cyanobacterial UV-screening extracellular phenolic pigment [57]. When proteins that synthesize the dialkylresorcinol (DAR) moiety are present (e.g., in *F. johnsoniae* cells), APEs are esterified with the DAR moiety and converted to flexirubin-type pigments (FTPs) (Fig. 5d). FTPs are well-studied yellow-to-orange pigments specific to Bacteroidetes and have been used as a chemosystematic marker for taxonomic studies because of its polyphyletic distribution [58, 59]. FTPs also absorb UV and visible light [59, 60] and localize to outer membranes [56], and can be detected by a flexirubin test [58]. Thus, we conducted a flexirubin test on *Aequorivita capsosiphonis* DSM 23843^T^, whose genome codes APE synthesis genes and the *darA* (ENOG08K4P) and *darB* (ENOG05CXX) genes that are used to synthesize the DAR moiety (Fig. 5a). This test experimentally confirmed that this strain actually synthesizes FTPs (data not shown). Another strain that has these genes, *Aquimarina muelleri* DSM 19832^T^, was also reported to respond positively to the flexirubin test [61].

Finally, the third cluster formed in the gene proximity network contained photolyase and photolyase-related genes and was enriched in the PR+ genomes (Fig. 4). Photolyase is an enzyme that uses visible light energy to repair DNA damage caused by UV light [62]. Specifically, the PR+ genomes coded significantly more genes of a photolyase paralogue (ENOG05CVP) than the PR− genomes did (Fig. 1, PR−: 1.9, PR+: 2.9 copies per genome on average).

## Discussion

**Fig. 6:**
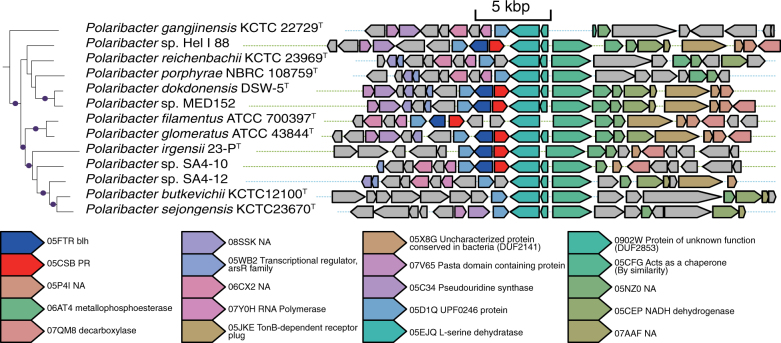
Syntenic map of the region around PR genes in the *Polaribacter* genomes. A part of the genomic phylogenetic tree is from Fig. 1. The closed circles indicate branches with 95% bootstrapping support. Blue and green horizontal dotted lines indicate PR− and PR+ strains, respectively. Pentagons represent genes, and pentagon lengths are proportional to the gene lengths. The conserved genes are shown in colours based on their annotation. Other genes are shown in grey. NA: Not Annotated. Because it is highly unlikely that the PR and *blh* genes were independently and repeatedly acquired next to the l-serine dehydratase gene by chance, the PR and *blh* genes are assumed to have been lost during evolution

In this study, we conducted a comparative genomic analysis of PR− and PR+ marine Flavobacteriia. The large and unbiased genomic data set enabled us to clarify their differences, which appear to be related to fundamentally different lifestyles and ecophysiological strategies. In addition, the polyphyletic distribution of PR genes (Fig. 1) and genomic traces indicated that PR genes have not only been gained but also lost during evolution (Fig. 6), suggesting that the conditions that have made each of the PR– and PR+ lifestyles advantageous have not been stable during the course of evolution. The approach adopted in this study can be further applied to provide broader insights into microbial ecology in the future—the more genomes we have, the more powerful comparative genomic approaches become.

**Fig. 7:**
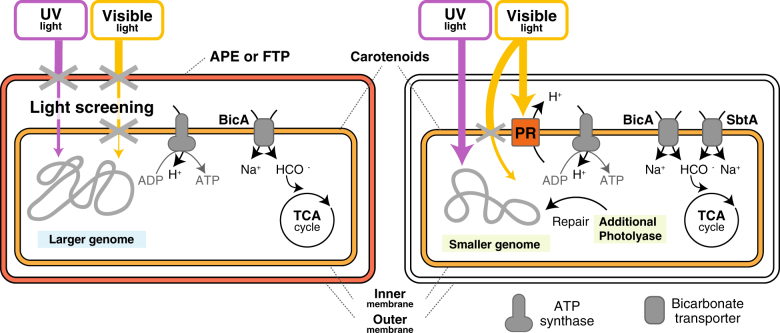
Schematic figure of the adaptive strategies of PR− and PR+ Flavobacteriia. The background colours in light blue and green represent characteristics of the PR− and PR+ marine Flavobacteriia, respectively. PR− strains have APEs or FTPs in the outer membrane to block UV and visible light. On the other hand, PR+ strains have neither APEs nor FTPs, but their PR can utilize visible light in the inner membrane. UV light that reaches the DNA in PR+ strains causes DNA damage, which is repaired by photolyases but leads to the smaller genome size of the PR+ strains. One gene that is involved in anaplerotic inorganic carbon fixation (*sbtA*) is biased in PR+ genomes. The PR− strains show signs of adaptation to anaerobic conditions

Although it might still be possible that the exclusive distribution pattern between the pigment synthesis and PR genes is due to unknown molecular mechanisms that prevent their co-existence, our results strongly suggest that PR− and PR+ marine Flavobacteriia adopt contrasting strategies to address UV damage: the former produces APEs or FTPs to avoid UV damage, whereas the latter produces photolyase to efficiently repair themselves after UV damage (Fig. 7). We propose that PR+ Flavobacteriia accept both UV damage and cost of repairing UV-damaged DNA so that they can take advantage of light energy by using PR in their inner membranes. On the other hand, PR− Flavobacteriia avoid the UV damage by blocking the UV light and thus must abandon utilizing light energy. To confirm the generality of our finding across different taxonomic groups, we analyzed the distribution patterns of rhodopsin and APE synthesis genes in all prokaryotes. While both rhodopsin and APE synthesis genes are distributed across diverse phyla, we observed their completely exclusive distribution patterns, that is, no strain possesses both rhodopsin and APE genes (Table 1). In accord with the analogy in which PR functions as microbial “solar panels”, we propose a “solar-panel or parasol” hypothesis, in which APEs and FTPs are regarded as cellular “parasols”. In this framework, we can choose to either charge solar-powered devices or use parasols to avoid tanning but cannot do both simultaneously.

**Table 1:** Numbers of genomes that code rhodopsin and APE synthesis genes

Taxonomic group	Rhodopsin+		Rhodopsin−	
	APE+	APE−	APE+	APE−
Bacteroidetes	0	15	33	125
Chloroflexi	0	1	0	10
Cyanobacteria	0	1	0	60
Deinococcusthermus	0	3	0	13
Firmicutes	0	2	0	690
Planctomycetes	0	1	0	6
Alphaproteobacteria	0	12	0	278
Betaproteobacteria	0	2	0	197
Gammaproteobacteria	0	10	0	730
Deltaproteobacteria	0	0	4	51
Sphingobacteriia	0	1	2	7

Genomes that code >50% of APE synthesis genes (eggNOG ID: 05C84, 05EWP, 05F6T, 05IAS, 05VME, 0636E, 07T6G, 08M6I, 05DZS, 05EYS, 05H6H, 05M49, 05YNY, 07T3I, 08H1G, and 08XF1) were regarded as APE-synthesizing (APE+) strains. The data were obtained from the eggNOG database version 4.5 [25]

Notably, these two different strategies for the handling of UV damage may also explain the smaller genome size of PR+ Flavobacteriia. First, UV damage itself would accelerate the net rate of genome size reduction in the PR+ strains via induced double-strand breaks and nonsense mutations [63]. Second, stronger selection pressure to minimize the DNA repair cost would also lead to the smaller genome size in PR+ Flavobacteriia. In contrast, PR− Flavobacteriia would receive less DNA damage and bear less cost for maintaining DNA; thus, they may be able to maintain a larger genome. It should be noted that bacteria in the SAR11 clade, which are the most abundant PR+ bacteria in the ocean, also have three copies of photolyase genes to repair damaged DNA despite their small genome sizes [64]. It may also be notable that deep-ocean SAR11 bacteria have larger genomes than those of surface-ocean SAR11 bacteria, which receive more UV damage [13]. Culture experiments for various strains that do and do not have pigment synthesis genes under different UV conditions will be required to further validate our hypothesis.

The evidence for the adaptation of PR− Flavobacteriia to conditions that are characterized by genes associated with anaerobic lifestyles provides another perspective on their ecophysiological adaptation (Fig. 7). Because molecular oxygen is required to synthesize retinal [65], PR+ bacteria are expected to prefer aerobic environments. To directly confirm this relationship between rhodopsins and oxygen, we re-analyzed the shotgun metagenomic data of Tara Oceans samples [36] and observed a positive correlation between rhodopsin gene abundance and oxygen concentration, even after normalizing for the effects of sampling depths (Pearson’s partial correlation = 0.61, *n* = 133) (Supplementary Fig. S6). Although Flavobacteriia are generally thought to be aerobic, it may be noted that a species in the family Flavobacteriaceae (*Muricauda ruestringensis* DSM 13258^T^) has nitrous oxide reductase genes (*nosZ* and *nosY*) and was reported to be facultative anaerobic [66]. We also note that the presence of Flavobacteriia is significant in environments with nanomolar oxygen concentrations and that nitrous oxide reductase genes are more abundant in particle-associated microbial communities than in free-living communities [67]. Thus, the interiors of macroscopic organic aggregates (also known as marine snows) in the upper ocean, which are known to be inhabited by Flavobacteriia [43, 68], are an environment where facultative anaerobic PR− microbes may predominate because their microaerobic (and nutrient-rich) conditions likely decrease the advantage of possessing PR [69]. Although the sampling sites of the strains analyzed in this study were not geographically comprehensive and did not show any geographic trend per the presence and absence of PR genes (Supplementary Fig. S1), we hypothesize that a possible geographic niche of facultative anaerobic PR– Flavobacteriia with UV protective pigments might be the surface layer in the eastern tropical north Pacific ocean, whose oxygen concentration is <10 µM even in the near-surface layer [70]. We envision that large-scale shotgun metagenomic analyses of macroscopic organic aggregates will be required to clarify this hypothesis.

## Electronic supplementary material

SI legends

Table S1. List of 21 marine Flavobacteriia genomes that were sequenced in this study

Table S2. List of 76 marine Flavobacteriia genomes. The isolation sites of the publicly available genome sequences were acquired from the IMG database (Markowitz et al 2013)

Table S3. Estimated origins of scaffolds of Polaribacter sejongensis KCTC 23670T and Polaribacter reichenbachii KCTC 23969T genomes

Table S4. List of eggNOG orthologue groups with distributions biased to PR− Flavobacteriia

Table S5. List of eggNOG orthologue groups with distributions biased to PR+ Flavobacteriia

Figure S1. Sampling sites of 54 flavobacterial strains whose geographical information was available

Figure S2. Phylogenetic tree of rhodopsin genes

Figure S3. Distributions of genes involved in anaplerotic inorganic carbon fixation

Figure S4. Phylogenetic tree of ribonucleotide reductase (RNR) genes

Figure S5. Distributions of cbb3-type cytochrome oxidase genes

Figure S6. Relationships between depth, oxygen concentration, and abundance of rhodopsin genes in the metagenomic data set of Tara Oceans samples
