# Supplementary material for: Solar-panel and parasol strategies shape the proteorhodopsin distribution pattern in marine Flavobacteriia
Source: ISME J. 2018 Feb 6;12(5):1329–43. doi: 10.1038/s41396-018-0058-4 (PMC5932025; doi:10.1038/s41396-018-0058-4)
Supplement: Supplementary file 1 — SI legends [file 41396_2018_58_MOESM1_ESM.pdf]

**Supporting information**

Tables are submitted as separated Excel files.

**Table S1.** List of 21 marine Flavobacteriia genomes that were sequenced in this study.

**Table S2.** List of 76 marine Flavobacteriia genomes.
The isolation sites of the publicly available genome sequences were acquired from the IMG database (Markowitz et al 2013)

**Table S3.** Estimated origins of scaffolds of *Polaribacter sejongensis* KCTC 23670$^T$ and *Polaribacter reichenbachii* KCTC 23969$^T$ genomes.

**Table S4.** List of eggNOG orthologue groups with distributions biased to PR－ Flavobacteriia.

**Table S5.** List of eggNOG orthologue groups with distributions biased to PR+ Flavobacteriia.

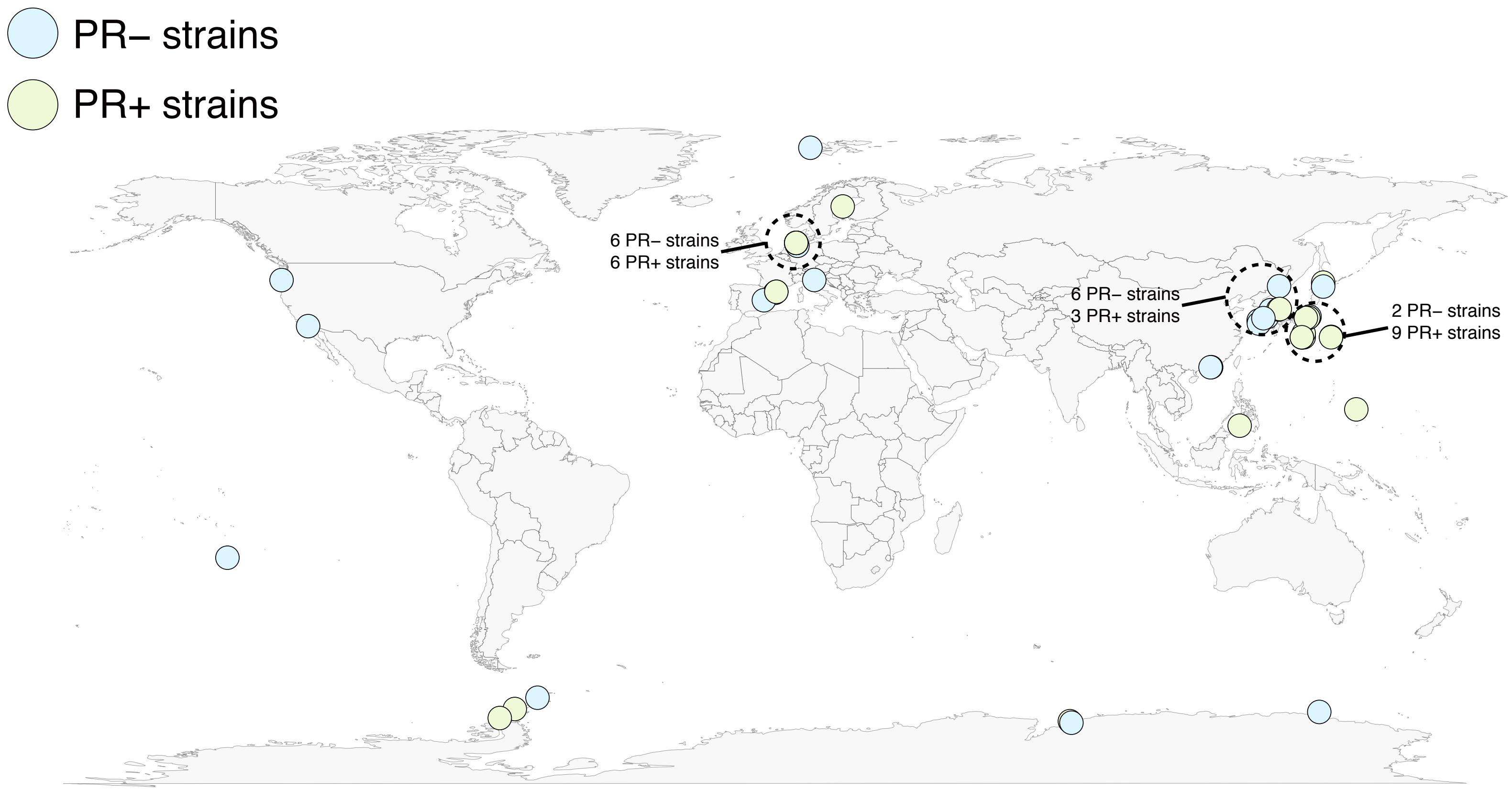


**Figure S1.** Sampling sites of 54 flavobacterial strains whose geographical information was available.
The centers of the circles indicate sampling sites (Yellow: PR–, Purple: PR+). The geographical (latitude and longitude) information was obtained from the IMG database (Markowitz et al 2013) except for the strains newly sequenced in this study. Note that the geographical information was not available for 22 of the 76 strains. This figure was generated using the *maps* package of R software (https://cran.r-project.org/web/packages/maps/maps.pdf).

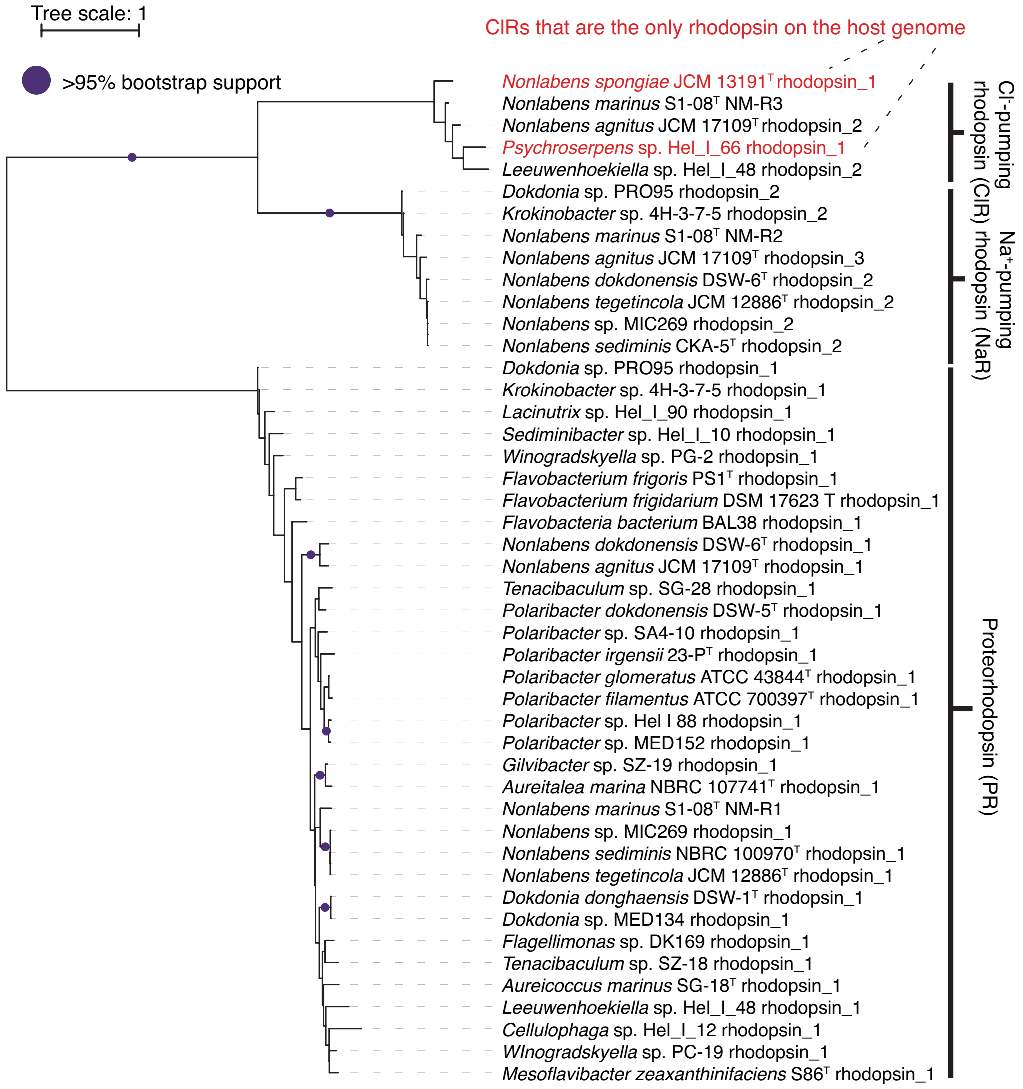


**Figure S2.** Phylogenetic tree of rhodopsin genes.

A maximum-likelihood tree of rhodopsin genes (CDSs annotated to bactNOG05CSB). The closed circles indicate branches with 95% bootstrapping support. Gene names in red indicate those genes whose genomes have $Cl^-$-pumping rhodopsin (ClR) genes as their only rhodopsin gene. The tree was visualized using iTol v 3.3.2.

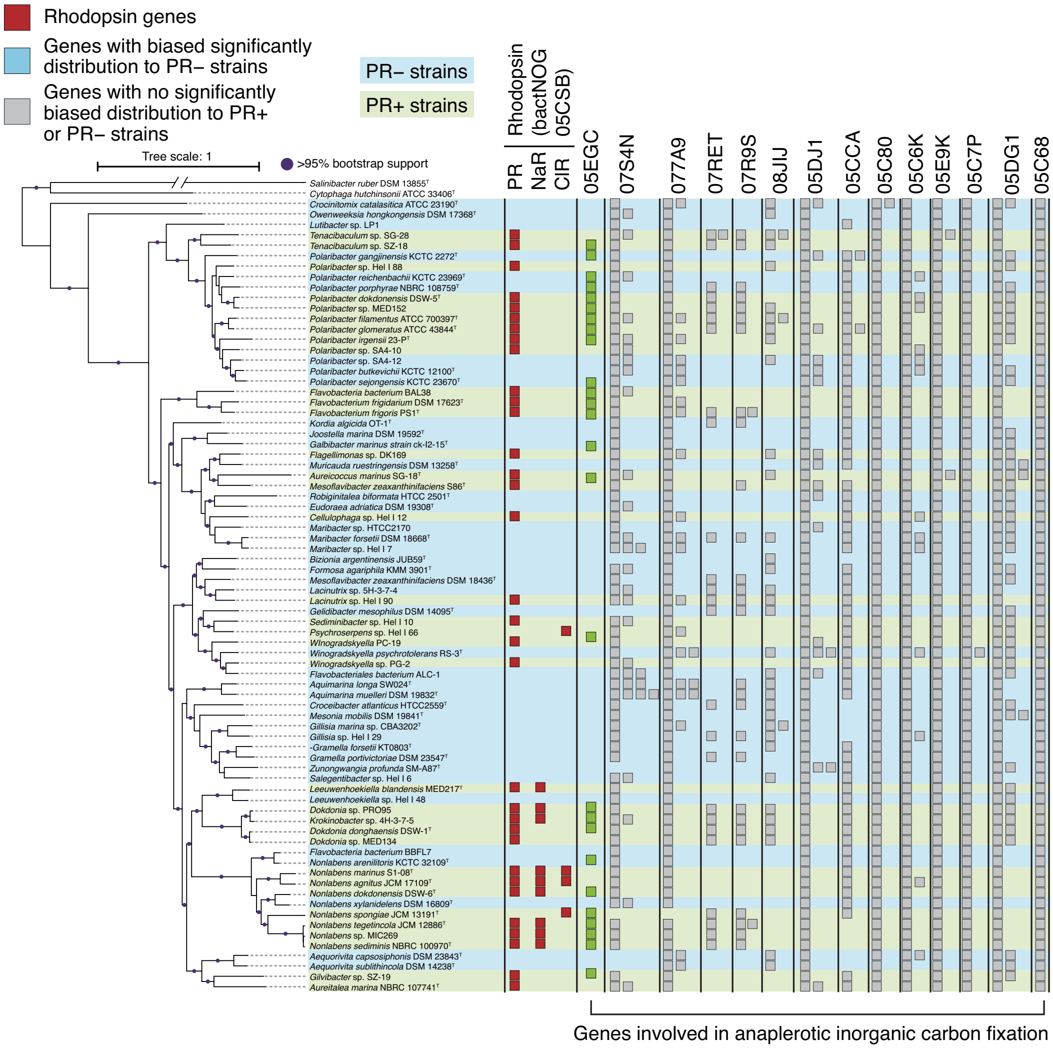


**Figure S3.** Distributions of genes involved in anaplerotic inorganic carbon fixation.

The genomic phylogenetic tree is from Fig. 1. The closed circles indicate branches with 95% bootstrapping support. Blue and green horizontal dotted lines indicate the PR− and PR+ strains, respectively. The number of genes encoded by each genome is illustrated by the number of closed squares. Red: rhodopsin genes. Light green and grey: bactNOG orthologue groups that are involved in anaplerotic inorganic carbon fixation (05EGC: *sbtA*, 07S4N: *bicA*, 077A9: carbonic anhydrase, 07RET: isocitrate lyase, 07R9S: malate synthase, 08JIJ: pyruvate carboxylase, 05DJ1: phosphoenolpyruvate carboxykinase, 05CCA: phosphoenolpyruvate carboxylase, 05C80: malate dehydrogenase, 05C6K: malate dehydrogenase, 05E9K: isocitrate dehydrogenase, and 05C7P: 2-oxoglutarate dehydrogenase subunit E1). The 05EGC group is coloured in light green because it showed distributions that were significantly biased to the PR+ genomes.

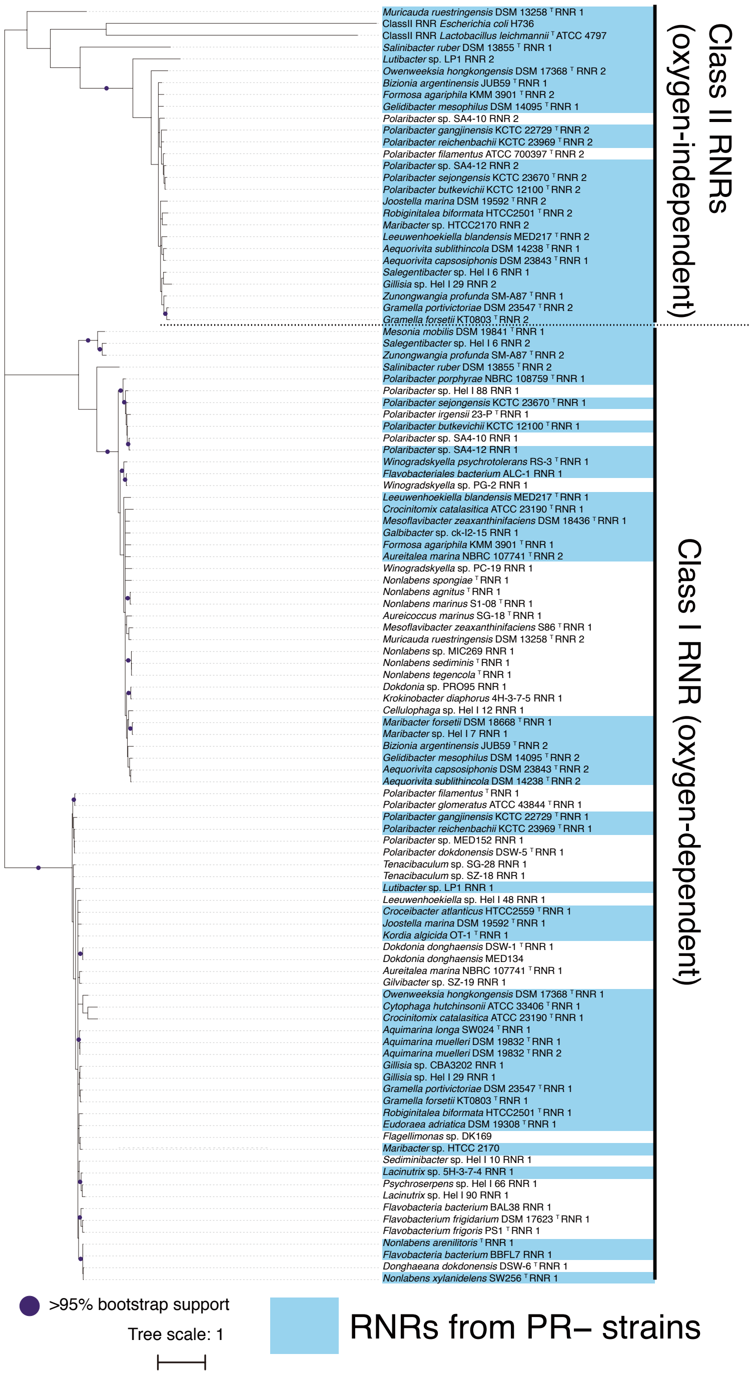


**Figure S4.** Phylogenetic tree of ribonucleotide reductase (RNR) genes.

The RNR classes, which are consistent with the phylogenetic tree organization, were identified by using an NCBI conserved domain search. The light blue background colour indicates those genes in PR－ genomes (otherwise, PR+ genomes). Any branch with less than 30% bootstrap support was removed, and those with more than 95% bootstrap support are represented by purple circles.

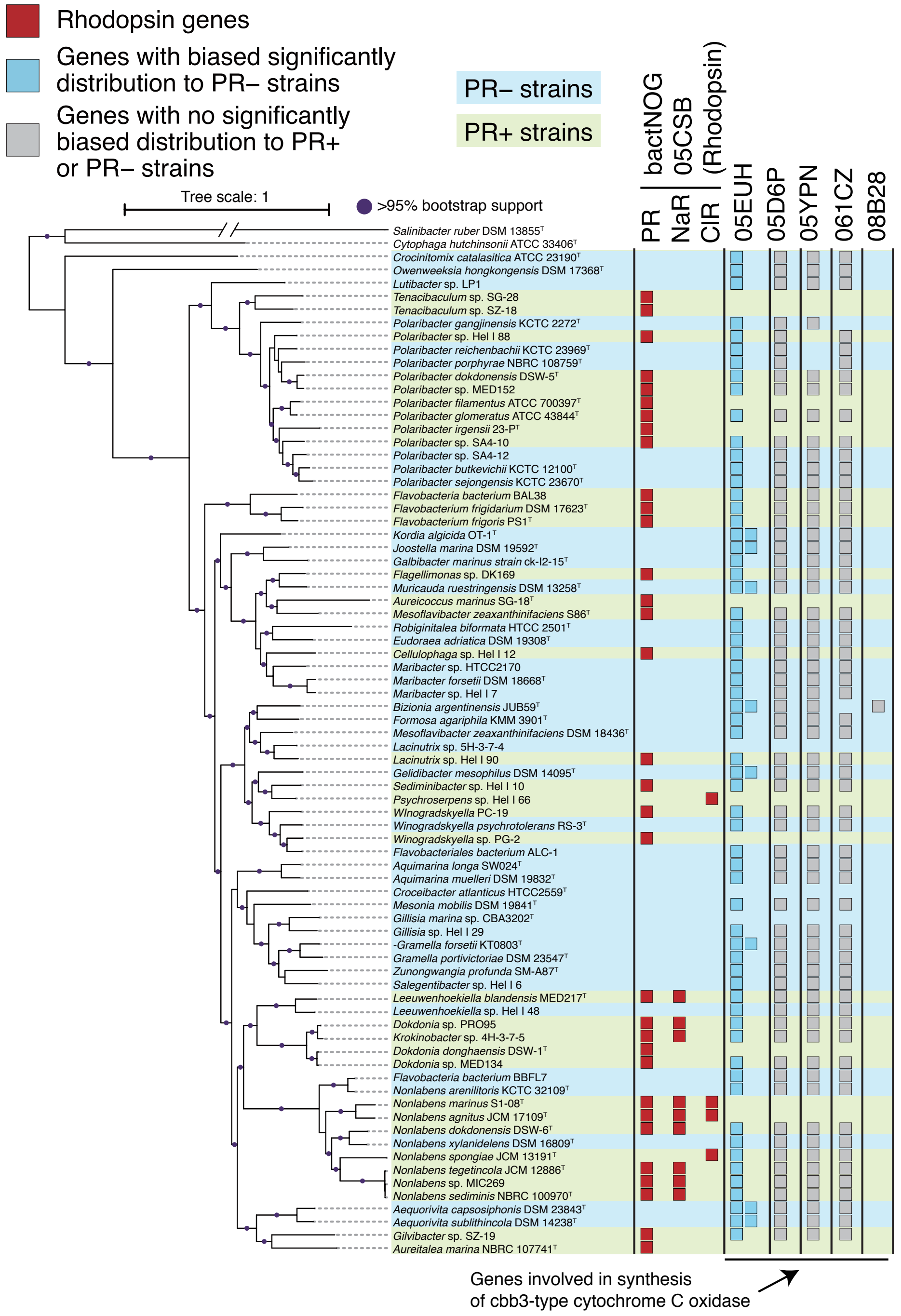


**Figure S5.** Distributions of *cbb*3-type cytochrome oxidase genes.

The genomic phylogenetic tree is from Fig. 1. The closed circles indicate branches with 95% bootstrapping support. Blue and green horizontal dotted lines indicate PR－ and PR+ strains, respectively. The number of genes encoded by each genome is illustrated by the number of closed squares. Red: rhodopsin genes. Light blue and grey: bactNOG orthologue groups of *cbb*3-type cytochrome oxidase genes (05EUH: *cbb*3-type cytochrome *c* oxidase subunit I, 05D6P: a *cbb*3-type cytochrome *c* oxidase complex protein, 05YPN: *cbb*3-type cytochrome oxidase maturation protein, 061CZ: *cbb*3-type cytochrome oxidase component FixQ, and 08B28: *cbb*3-type cytochrome oxidase component FixQ). The 05EUH group is coloured in light blue because it showed distributions that were significantly biased to the PR– genomes.

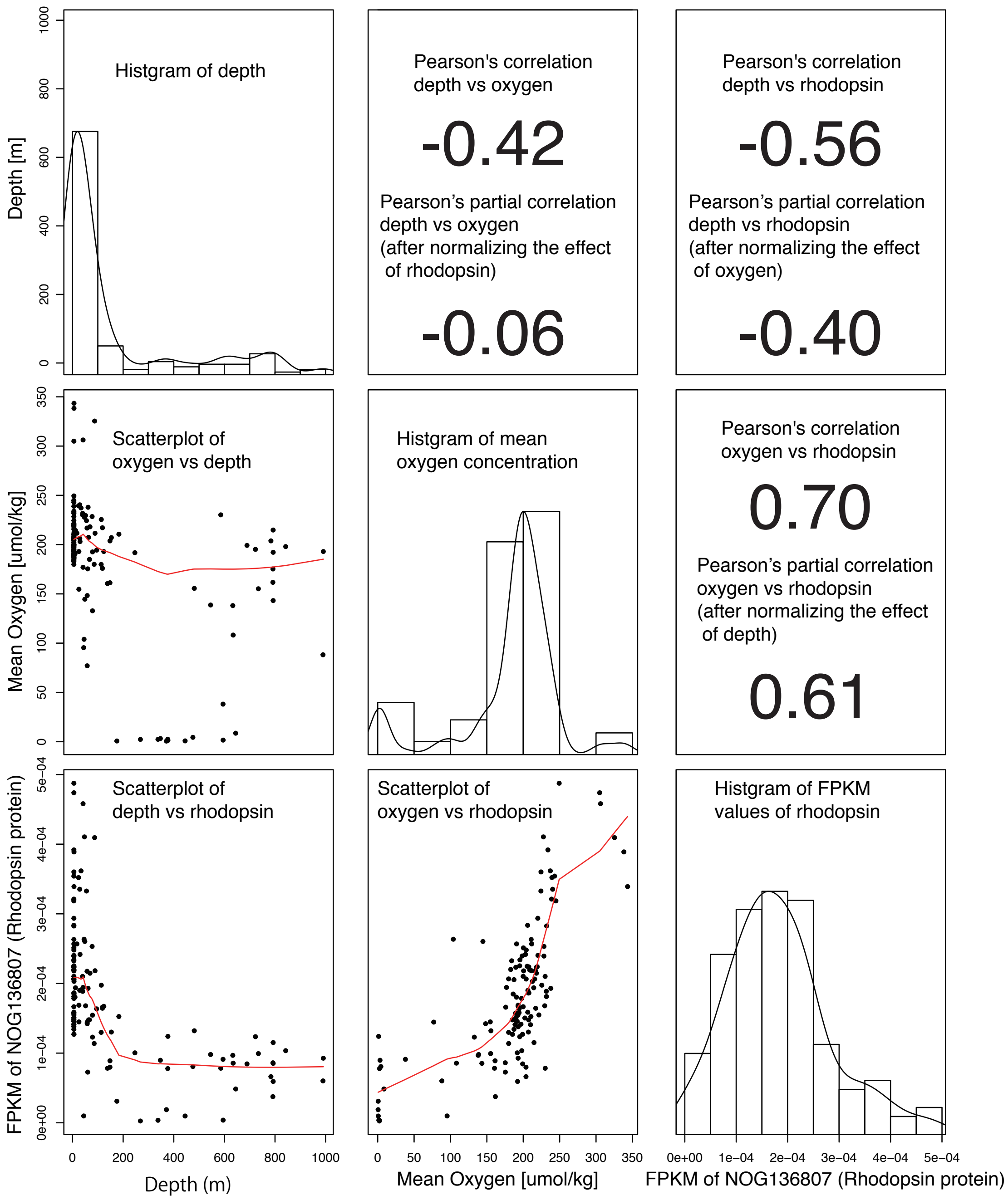


**Figure S6.** Relationships between depth, oxygen concentration, and abundance of rhodopsin genes in the metagenomic dataset of Tara Oceans samples.

The rhodopsin gene abundances were quantified by their Fragments Per Kilobase of exon per Million fragments (FPKM) values. Histograms of the three values, scatterplots, Pearson's correlation values, and Pearson's partial correlation values are shown.

**Supporting information references**

Markowitz VM, Chen I-MA, Palaniappan K, Chu K, Szeto E, Pillay M *et al* (2013). IMG 4 version of the integrated microbial genomes comparative analysis system. *Nucleic Acids Res* **42:** D560-D567.
