# Supplementary figures and images for: Solar-panel and parasol strategies shape the proteorhodopsin distribution pattern in marine Flavobacteriia
Source: ISME J. 2018 Feb 6;12(5):1329–43. doi: 10.1038/s41396-018-0058-4 (PMC5932025; doi:10.1038/s41396-018-0058-4)

PR- strains

PR+ strains

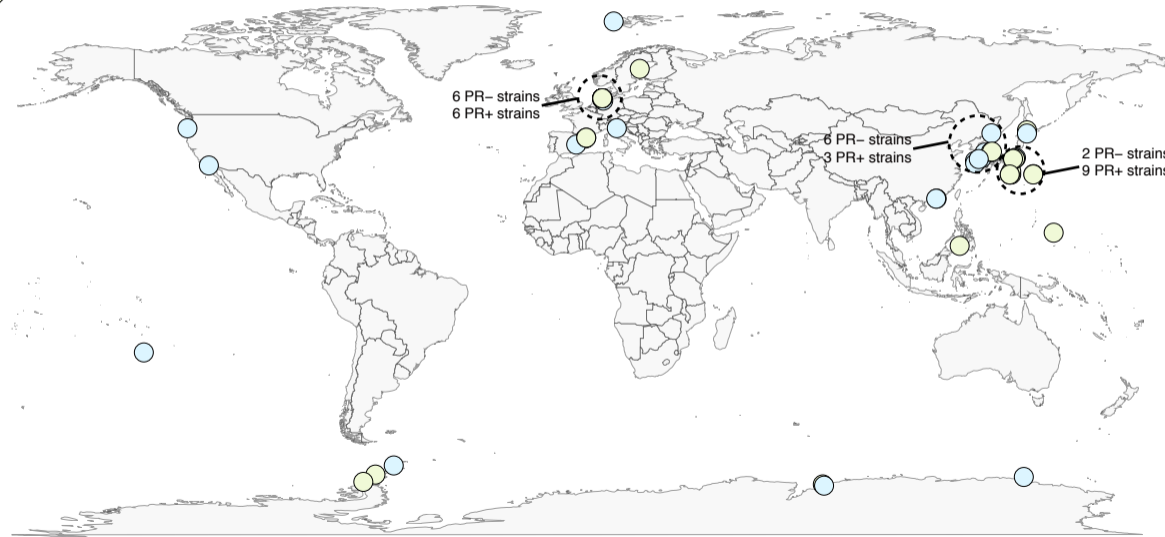

Supplement: Supplementary file 7 — Figure S1. Sampling sites of 54 flavobacterial strains whose geographical information was available [file 41396_2018_58_MOESM7_ESM.pdf]

PR+ strains

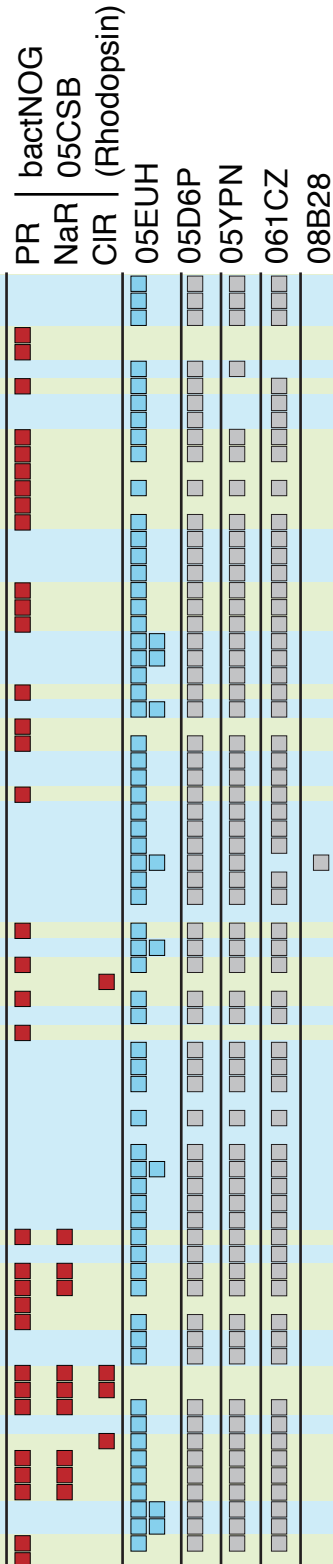

Supplement: Supplementary file 11 — Figure S5. Distributions of cbb3-type cytochrome oxidase genes [file 41396_2018_58_MOESM11_ESM.pdf]
