# Supplementary material for: Solar-panel and parasol strategies shape the proteorhodopsin distribution pattern in marine Flavobacteriia
Source: ISME J. 2018 Feb 6;12(5):1329–43. doi: 10.1038/s41396-018-0058-4 (PMC5932025; doi:10.1038/s41396-018-0058-4)
Supplement: Supplementary file 10 — Figure S4. Phylogenetic tree of ribonucleotide reductase (RNR) genes [file 41396_2018_58_MOESM10_ESM.pdf]

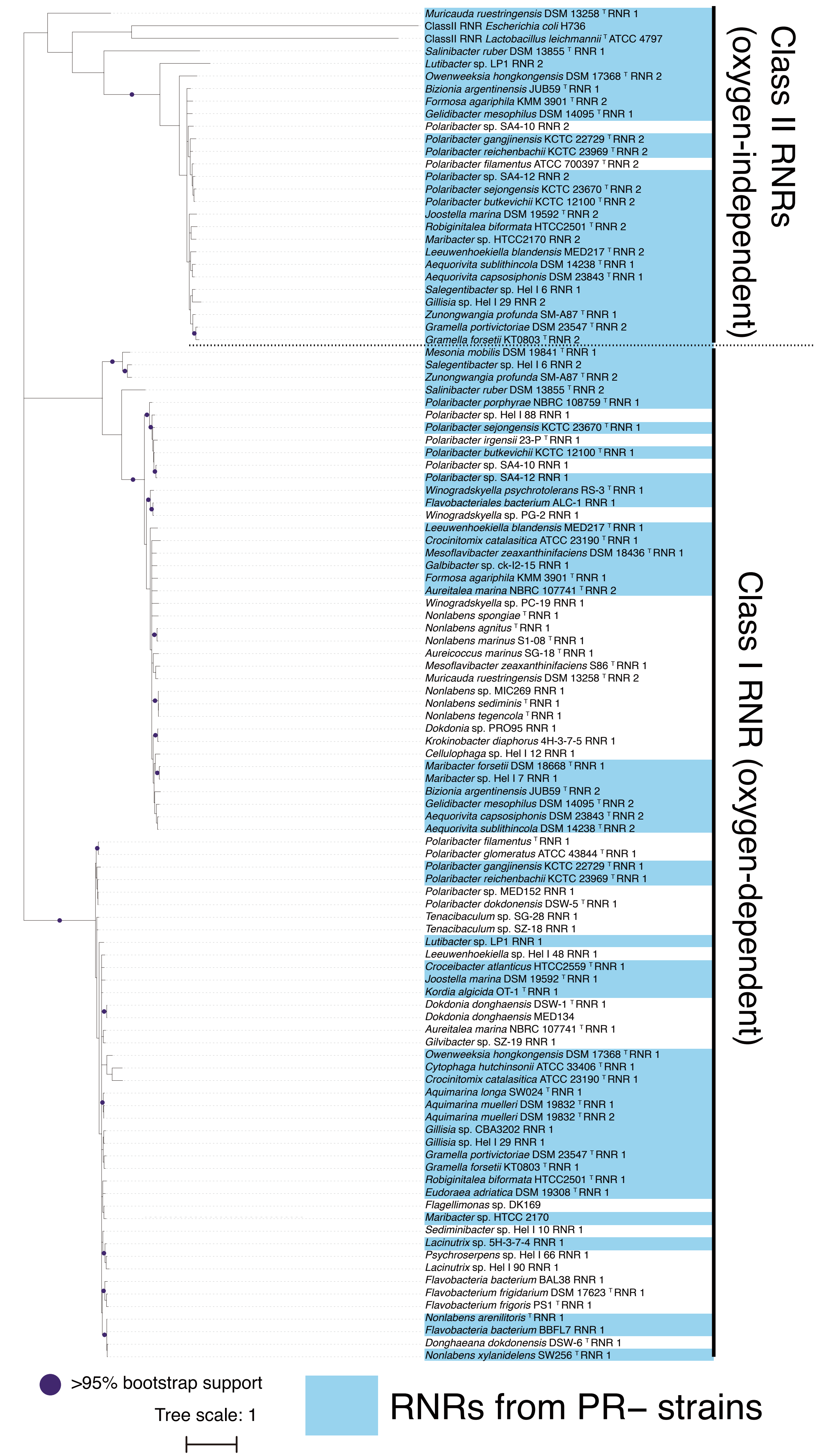
Class II RNRs (oxygen-independent)
Class I RNR (oxygen-dependent)
Muricauda ruestringensis DSM 13258 T RNR 1
ClassII RNR Escherichia coli H736
ClassII RNR Lactobacillus leichmannii T ATCC 4797
Salinibacter ruber DSM 13855 T RNR 1
Lutibacter sp. LP1 RNR 2
Owenweeksia hongkongensis DSM 17368 T RNR 2
Bizionia argentinensis JUB59 T RNR 1
Formosa agariphila KMM 3901 T RNR 2
Gelidibacter mesophilus DSM 14095 T RNR 1
Polaribacter sp. SA4-10 RNR 2
Polaribacter gangjinensis KCTC 22729 T RNR 2
Polaribacter reichenbachii KCTC 23969 T RNR 2
Polaribacter filamentus ATCC 700397 T RNR 2
Polaribacter sp. SA4-12 RNR 2
Polaribacter sejongensis KCTC 23670 T RNR 2
Polaribacter butkevichii KCTC 12100 T RNR 2
Joostella marina DSM 19592 T RNR 2
Robiginitalea biformata HTCC2501 T RNR 2
Maribacter sp. HTCC2170 RNR 2
Leeuwenhoekiella blandensis MED217 T RNR 2
Aequorivita sublithincola DSM 14238 T RNR 1
Aequorivita capsosiphonis DSM 23843 T RNR 1
Salegentibacter sp. Hel I 6 RNR 1
Gillisia sp. Hel I 29 RNR 2
Zunongwangia profunda SM-A87 T RNR 1
Gramella portivictoriae DSM 23547 T RNR 2
Gramella forsetii KT0803 T RNR 2
Mesonia mobilis DSM 19841 T RNR 1
Salegentibacter sp. Hel I 6 RNR 2
Zunongwangia profunda SM-A87 T RNR 2
Salinibacter ruber DSM 13855 T RNR 2
Polaribacter porphyrae NBRC 108759 T RNR 1
Polaribacter sp. Hel I 88 RNR 1
Polaribacter sejongensis KCTC 23670 T RNR 1
Polaribacter irgensii 23-P T RNR 1
Polaribacter butkevichii KCTC 12100 T RNR 1
Polaribacter sp. SA4-10 RNR 1
Polaribacter sp. SA4-12 RNR 1
Winogradskyella psychrotolerans RS-3 T RNR 1
Flavobacteriales bacterium ALC-1 RNR 1
Winogradskyella sp. PG-2 RNR 1
Leeuwenhoekiella blandensis MED217 T RNR 1
Crocinitomix catalasitica ATCC 23190 T RNR 1
Mesoflavibacter zeaxanthinifaciens DSM 18436 T RNR 1
Galbibacter sp. ck-I2-15 RNR 1
Formosa agariphila KMM 3901 T RNR 1
Aureitalea marina NBRC 107741 T RNR 2
Winogradskyella sp. PC-19 RNR 1
Nonlabens spongiae T RNR 1
Nonlabens agnitus T RNR 1
Nonlabens marinus S1-08 T RNR 1
Aureicoccus marinus SG-18 T RNR 1
Mesoflavibacter zeaxanthinifaciens S86 T RNR 1
Muricauda ruestringensis DSM 13258 T RNR 2
Nonlabens sp. MIC269 RNR 1
Nonlabens sediminis T RNR 1
Nonlabens tegencola T RNR 1
Dokdonia sp. PRO95 RNR 1
Krokinobacter diaphorus 4H-3-7-5 RNR 1
Cellulophaga sp. Hel I 12 RNR 1
Maribacter forsetii DSM 18668 T RNR 1
Maribacter sp. Hel I 7 RNR 1
Bizionia argentinensis JUB59 T RNR 2
Gelidibacter mesophilus DSM 14095 T RNR 2
Aequorivita capsosiphonis DSM 23843 T RNR 2
Aequorivita sublithincola DSM 14238 T RNR 2
Polaribacter filamentus T RNR 1
Polaribacter glomeratus ATCC 43844 T RNR 1
Polaribacter gangjinensis KCTC 22729 T RNR 1
Polaribacter reichenbachii KCTC 23969 T RNR 1
Polaribacter sp. MED152 RNR 1
Polaribacter dokdonensis DSW-5 T RNR 1
Tenacibaculum sp. SG-28 RNR 1
Tenacibaculum sp. SZ-18 RNR 1
Lutibacter sp. LP1 RNR 1
Leeuwenhoekiella sp. Hel I 48 RNR 1
Croceibacter atlanticus HTCC2559 T RNR 1
Joostella marina DSM 19592 T RNR 1
Kordia algicida OT-1 T RNR 1
Dokdonia donghaensis DSW-1 T RNR 1
Dokdonia donghaensis MED134
Aureitalea marina NBRC 107741 T RNR 1
Gilvibacter sp. SZ-19 RNR 1
Owenweeksia hongkongensis DSM 17368 T RNR 1
Cytophaga hutchinsonii ATCC 33406 T RNR 1
Crocinitomix catalasitica ATCC 23190 T RNR 1
Aquimarina longa SW024 T RNR 1
Aquimarina muelleri DSM 19832 T RNR 1
Aquimarina muelleri DSM 19832 T RNR 2
Gillisia sp. CBA3202 RNR 1
Gillisia sp. Hel I 29 RNR 1
Gramella portivictoriae DSM 23547 T RNR 1
Gramella forsetii KT0803 T RNR 1
Robiginitalea biformata HTCC2501 T RNR 1
Eudoraea adriatica DSM 19308 T RNR 1
Flagellimonas sp. DK169
Maribacter sp. HTCC 2170
Sediminibacter sp. Hel I 10 RNR 1
Lacinutrix sp. 5H-3-7-4 RNR 1
Psychroserpens sp. Hel I 66 RNR 1
Lacinutrix sp. Hel I 90 RNR 1
Flavobacteria bacterium BAL38 RNR 1
Flavobacterium frigidarium DSM 17623 T RNR 1
Flavobacterium frigoris PS1 T RNR 1
Nonlabens arenilitoris T RNR 1
Flavobacteria bacterium BBFL7 RNR 1
Donghaeana dokdonensis DSW-6 T RNR 1
Nonlabens xylanidelens SW256 T RNR 1
>95% bootstrap support
Tree scale: 1
RNRs from PR– strains
